# Stroking or Buzzing? A Comparison of Somatosensory Touch Stimuli Using 7 Tesla fMRI

**DOI:** 10.1371/journal.pone.0134610

**Published:** 2015-08-18

**Authors:** Wietske van der Zwaag, Rolf Gruetter, Roberto Martuzzi

**Affiliations:** 1 Centre d’Imagerie Biomédicale (CIBM), École Polytechnique Fédérale de Lausanne (EPFL), Lausanne, Switzerland; 2 Spinoza Centre for Neuroimaging, Royal Netherlands Academy of Arts and Sciences (KNAW), Amsterdam, Netherlands; 3 Department of Radiology, University Hospital, Lausanne, Switzerland; 4 Department of Radiology, University Hospital, Geneva, Switzerland; 5 Center for Neuroprosthetics, School of Life Sciences, Ecole Polytechnique Fédérale de Lausanne (EPFL), Lausanne, Switzerland; 6 Laboratory of Cognitive Neuroscience, Brain Mind Institute, Ecole Polytechnique Fédérale de Lausanne (EPFL), Lausanne, Switzerland; 7 Fondation Campus Biotech Geneva, Geneva, Switzerland; Indiana University, UNITED STATES

## Abstract

Studying body representations in the brain helps us to understand how we humans relate to our own bodies. The *in vivo* mapping of the somatosensory cortex, where these representations are found, is greatly facilitated by the high spatial resolution and high sensitivity to brain activation available at ultra-high field. In this study, the use of different stimulus types for somatotopic mapping of the digits at ultra-high field, specifically manual stroking and mechanical stimulation, was compared in terms of sensitivity and specificity of the brain responses. Larger positive responses in digit regions of interest were found for manual stroking than for mechanical stimulation, both in terms of average and maximum t-value and in terms of number of voxels with significant responses to the tactile stimulation. Responses to manual stroking were higher throughout the entire post-central sulcus, but the difference was especially large on its posterior wall, i.e. in Brodmann area 2. During mechanical stimulation, cross-digit responses were more negative than during manual stroking, possibly caused by a faster habituation to the stimulus. These differences indicate that manual stroking is a highly suitable stimulus for fast somatotopic mapping procedures, especially if Brodmann area 2 is of interest.

## Introduction

The human primary sensory cortex (SI) is well known to contain a body representation in which each part of the human body is represented by a specific brain region [1]. The body representations span several Brodmann Areas (BAs), in anterior-to-posterior order: BA3b on the anterior flank of the post-central sulcus, BA1 on its crown and BA2 on the posterior wall. BA3b is assumed to be the most primary of these, with BA1 and BA2 containing neurons with larger receptive fields involved in more complex processing. This agrees with the smaller overlap between digit representations in BA3b than in BA1 and BA2 [2–4]. The visualization of this somatosensory body representation with functional MRI (fMRI) requires high sensitivity to the somatosensory BOLD responses and high spatial resolution of the fMRI data, as well as a reliable somatosensory stimulus. Ultra-high field fMRI [5] provides both the required high spatial resolution and high BOLD sensitivity [6,7].

Over the last decade, somatotopy experiments have been conducted using a range of different stimulus types. For the finger tips, which form the easiest body regions to map because of their large brain representation and good physical access (unlike, for example, the lips, which are difficult to access in the scanner), piezo-electric devices, pneumatically driven stimulators, air-puffs, electrical stimulation and manually operated stroking tools have all been suggested. All of these stimulation methods have been employed more or less successfully to distinguish responses from the different fingers in at least BA 3b, where the digit representations are best separated [2–4]. Stimulation may or may not be accompanied by a task such as oddball detection [8] or a one-back task [9], to direct attention to the somatosensory modality.

The most popular method for finger mapping is the use of piezo-electric devices. These devices can generate vibrations over a large range of frequencies and can easily be computer-controlled from outside the scanner room, generating reproducible stimulation over any duration from a few hundred milliseconds to several seconds. Although the BOLD responses are known to vary as a function of stimulus frequency [10,11], the range of used stimulus frequencies is relatively wide (in somatosensory mapping 16 - >150 Hz). At a frequency of around 30 Hz a sense of flutter is elicited which is transmitted by the Meissner corpuscles [12], and the clear tactile sensation related with this stimulus frequency translates in reliable BOLD responses, resulting in several studies reporting results with a stimulus frequency of 30–32 Hz [13–16]. However, both lower stimulus frequencies, of 16 Hz [17] and 23 Hz [18], and higher stimulus frequencies (>50Hz), for example of 50 Hz [2,19], 80 Hz [10], 100 Hz [20] and 150 Hz [21] have also been used. Stimuli are often presented in an intermittent fashion, with short bursts quickly succeeding one another within a longer stimulus block, to avoid adaptation [14,16,20].

Pneumatic devices have also been suggested for use in the MR environment [22,23]. They are computer controllable and can be easily made MR compatible, but have led to less wide-spread adoption than that of piezo-electric devices, perhaps because of the reduced range of achievable frequencies (1–20 Hz; [22]). The specific case of using airpuffs as tactile stimulus in somatotopy mapping has been reported more often [8,24–26], with frequencies being used in the 2–10 Hz range. Airpuffs are more difficult to control in terms of timing, force amplitude and the exact location, but benefit from their simplicity and excellent scanner compatibility.

Another approach to stimulate SI consists of the direct electrical stimulation of the median nerve. This method has been shown to lead to robust BOLD responses in SI [27–29] but has the significant drawback of invalidating the non-invasive nature of fMRI and is thus typically used only for specific research questions. Stimulus clarity and strength also depend somewhat on the exact positioning of the electrode, demanding the presence of specifically trained and qualified personnel during the experiment. Cutaneous non-painful electrical stimulation (7Hz) has been used for inter- and intra-digit somatotopy [30,31], as well as for investigating the overlap between digit representations in the different Brodmann areas in SI [4].

Finally, the use of manually operated stroking tools or even direct human touch to the digits has also been shown to result in reliable somatotopic maps. Human-operated devices, usually a fine paintbrush [32], toothbrush [33], or touch [3,34,35] cannot be controlled automatically and rely on the transmission of cues to the operator. A large advantage is that the human touch is a strong and highly salient stimulus, which activates not only BA 3b but also BA 1 and 2 [3] and the cerebellar lobules V and VIII [34] sufficiently for somatotopic mapping in individual subjects. In addition, somatotopic mapping strategies using these stimulation methods can more easily be transferred to studies on the role of SI in cognitive and social tasks, which often use human touch as a stimulus [35–37].

To date, there are no studies systematically comparing multiple types of stimulation in humans. In rats, BOLD responses arising as a result from airpuffs deflecting whiskers has been compared to the application of an electrical current to the forepaw, showing a frequency dependence for both stimulation methods [11]. In this study, we investigated whether and how the choice of stimulus, i.e. human touch and mechanical stimulation, affects the results of high-resolution somatotopic mapping procedures with fMRI in terms of sensitivity and specificity of the BOLD responses. We hypothesized that the obtained somatotopic maps would be highly similar for the two stimulation methods, possibly with more focused BOLD responses for the mechanical stimulation.

## Methods

### Ethics statement

All subjects provided written informed consent and the study was approved by the Human Research Ethics Committee of the canton Vaud.

### Data acquisition

Eight right-handed volunteers (2 female, aged 22–28 years) participated in this experiment. Data were collected at a short-bore 7 T MR system (Siemens, Germany) with a head-gradient insert. A 32-channel receive array coil was used for rf-reception [38]. Three functional runs were acquired for each volunteer: a ‘mechanical stimulation’ run during which stimulation was applied with a piezo-electric device (MAG design & engineering), a ‘brush’ run during which a commercially available toothbrush was used and a ‘stroking’ run in which human touch was applied. During the entire experiment, participants lied in supine position in the scanner with their right arm comfortably stretched along the magnet bore. Data from one volunteer were excluded as due to time constraints the ‘brush’ run could not be acquired. The order of ‘brush’ and ‘stroking’ run acquisitions was counterbalanced across subjects.

During the ‘brush’ and ‘stroking’ runs, an experimenter was positioned at the entrance of the bore where he could easily reach and stroke the two distal phalanges of each digit with the toothbrush (‘brush’ run) or his own index finger (‘stroking’ run). The experimenter received auditory cues delivered on MR-compatible headphones (Nordic Neuro Lab, Norway), sent from a local pc using E-prime (Psychology Software Tools, PA). Each digit was independently stroked for 10 s, followed by 10 s of rest (no stroking). During the 10 s of stimulation, the two distal phalanges of each finger were repeatedly stroked along the axis of the finger, from the proximal to the distal portion of the finger at a frequency of approximately 1 Hz. During the ‘mechanical stimulation’ run, the piezo-electric device was controlled through E-Prime and activated in blocks of 10 s followed by 10 s of rest (no stimulation). During the 10 s block of activation, a 30 Hz pulsed stimulus (500 ms on, 500 ms off) was applied to the tip of the digit. At placement of the stimulators, care was taken to ensure that the 30Hz stimulus was perceived only on the digit tip and did not spread to other portions of the hand. To increase participants’ attention during mechanical stimulation, subjects were asked to detect and count oddball stimuli (500 ms pulse at 60 Hz, 5%). For the ‘stroking’ and ‘brush’ runs no additional task was necessary to engage subjects’ sustained attention. For each of the three runs the order in which the digits were stimulated was the same across runs: D1 (thumb), D3 (middle), D5 (little), D2 (index), D4 (ring). This sequence was repeated 8 times, resulting in a scan time of 13 minutes 20 seconds per run.

All fMRI data were acquired using an EPI sequence with sinusoidal readout, with 1.3x1.3x1.3mm^3^ resolution and TR/TE/α = 2000ms/27ms/75^0^, matrix size 160x160, 28 slices (no gap). The near-axial slices were placed perpendicular to the central sulcus and acquired in an interleaved fashion. The readout bandwidth was 1042 Hz/pixel, and a GRAPPA undersampling factor of 2 was used to limit the echo planar readout length and susceptibility induced distortions. 405 volumes were acquired per functional run.

Whole-brain anatomical data were acquired using the MP2RAGE sequence [39] with the following parameters: voxel size: 1x1x1 mm^3^, matrix size: 256 x 256 x 176, TR_MP2RAGE_ = 5500ms, TE = 2.82 ms, TI1/TI2 = 750/2350ms, GRAPPA factor 3, total acquisition time 7.27 minutes. To aid with the co-registration between the fMRI data and the anatomical volumes, a single whole-brain echo planar volume with 71 slices (placed with the same inclination as the functional EPI) and TR = 5000ms; all other scan parameters were the same as for the functional data

### Data analysis

FMRI data processing was carried out using SPM8 and routines written in Matlab (Mathworks, Inc.). Preprocessing steps included: joint motion correction of the three functional runs, spatial smoothing with a Gaussian of FWHM = 2mm and co-registration of the anatomical to the mean of the fMRI runs via the whole-brain echo planar volume. A GLM analysis was run for the three functional runs separately in which the model included the stimulation blocks of the five digits convolved with the hemodynamic response function (HRF) and their temporal derivatives; motion parameters were also included in the model as regressors of no interest. For each run five t-contrasts were generated, corresponding to stimulation of each digit vs rest.

**Fig 1:**
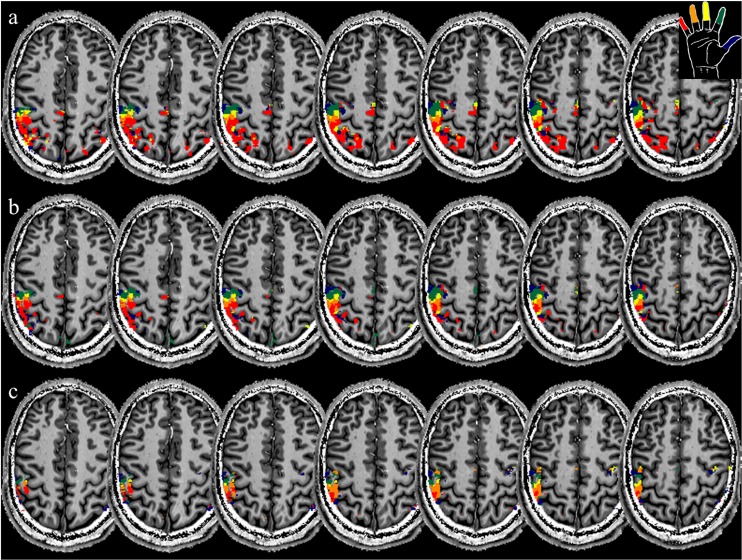
Comparison of label maps from an example subject, each generated from one functional run. a) Manual stroking. b) Brush stroking. c) Mechanical stimulation. Note the excellent spatial correspondence between the three maps. Although the ‘stroking’ and ‘brush’ runs yield much larger regions above threshold (p<0.001), the label identities in the above-threshold voxels agrees very well. 53% of all voxel in the overlapping region had the same digit label in all three maps; a further 24% of voxels were assigned to an ‘adjacent’ digit in one of the maps (i.e. 2-2-3).

**Fig 2:**
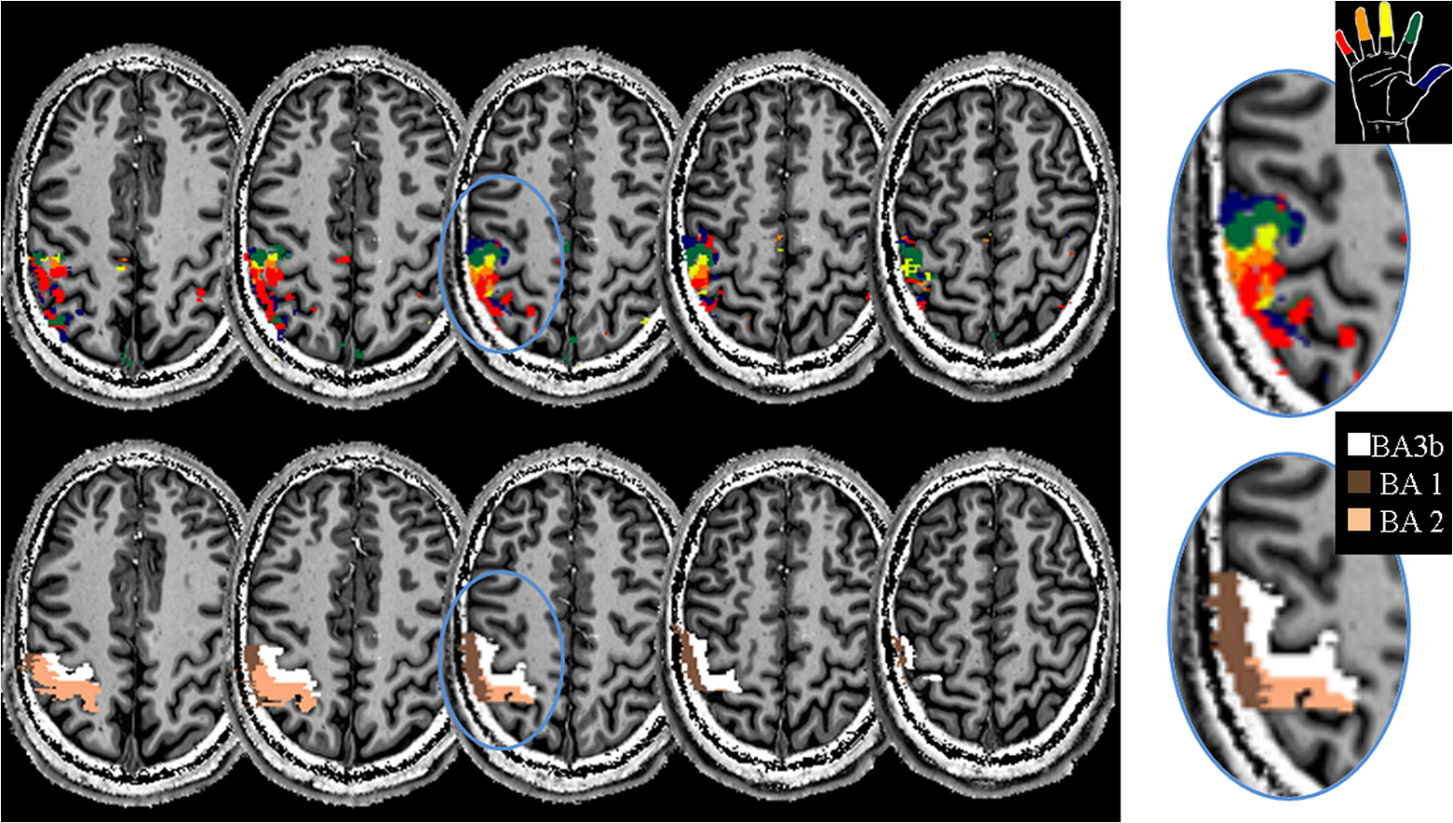
An example of a set of ROI definitions. The top row shows the labelling map generated from the ‘brush’ run. The bottom row shows the separation of the postcentral sulcus in anterior wall, crown, and posterior wall, or BA3b, BA1 and BA2, respectively. BA-ROIs were drawn on the mean echo planar image. Digit ROIs were generated for each BA separately by masking the labelling map. The blue circle indicates the region shown in the round panels. Note the full series of digit regions in each BA for this subject. While BA3b and BA1 contain an orderly series (D5-D4-D3-D2-D1 along the sulcus), the pattern in BA2 is not clearly visible as it spans several slices.

Definition of independent regions of interest (ROIs) requires acquisition of a separate functional run. Here, the ‘brush’ run was used only for the definition of the ROIs, because the brush stimulus has several features in common with both manual stroking and mechanical stimulation. In ‘stroking’ and ‘brush’ runs the stimulus irregularity and stimulated cutaneous surface area are the same, while in ‘brush’ and ‘mechanical stimulation’ runs stimulation was delivered using an object that does not convey body warmth information. A label map was generated from the ‘brush’ maps from an inclusive mask at p<0.001 of all five digit regressors (Fig 1B, top row Fig 2). Within the mask, voxels were assigned to the digit with the highest t-value in a winner-takes-all manner (see also: [3,34]). The ROIs were then generated by separating the label map in BA 3b, BA 1 and BA 2 regions using three manually generated masks drawn on the mean of the fMRI image, separating the flanks and crown of the post-central gyrus (Fig 2, bottom row). The anterior wall, crown and posterior wall are roughly corresponding to BAs 3b, 1 and 2, [40–42] and the ROIs will be referred to as such in the rest of this manuscript.

To compare average BOLD response amplitudes within the ROIs for the different stimulus types we computed mean beta values and mean t-values per ROI. To test for changes in the distribution of t-values, we measured maximum t-values and also compared the number of voxels showing responses to stimulation of the relevant digit with p<0.05 FWE corrected. In addition, the beta-values of cross-digit responses were investigated to estimate overlap between BOLD responses. In this context, responses to stimulation of the digit matching the ROI label are referred to as ‘matching’ digit responses, responses to stimulation of digits neighboring that of the digit ROI (for example changes in ROI1 during stimulation of D2) are referred to as ‘adjacent’ digit responses and responses to digits further removed are ‘distant’ responses. ‘Adjacent’ and ‘distant’ together are referred to as ‘cross-digit’ responses. For an illustration, see Table 1.

**Table 1:** Matching, adjacent and distant responses per ROI.

	Stimulation D1	D2	D3	D4	D5
ROI 1	**M**	A	D	D	D
ROI 2	A	**M**	A	D	D
ROI 3	D	A	**M**	A	D
ROI 4	D	D	A	**M**	A
ROI 5	D	D	D	A	**M**

Terminology for the BOLD responses to stimulation of a certain digit in the different digit ROIs. M = matching, A = adjacent, D = distant. Adjacent and distant responses are jointly referred to as cross-digit responses

To further characterize the t-value distribution within ROIs, the t-values for the ‘matching’ digit stimulation were extracted voxel-by-voxel and histograms of t-value distributions were generated with a bin-width of 0.2, ranging from -8 to 19 to catch all outliers. The t-value distributions were fitted with a t-distribution function (Eq 1) with variables scale S, mean μ and width σ, to study differences in distribution width.

$$y=\frac{S}{\sqrt{\nu \sigma^{2}}B\left(\frac{\nu }{2},\frac{1}{2}\right)}{\left(1+\frac{{\left(x-\mu \right)}^{2}}{\nu \sigma^{2}}\right)}^{-\frac{\nu +1}{2}}$$ (1)

Y is the distribution of t-values and x the centre value of the bins, ν is the degrees of freedom (number of voxels included in the histogram) and B is the beta function. A non-linear least squares fit in Matlab was used for both the overall distribution and the subject-by-subject distributions. Differences in μ and σ were tested with a paired t-test.

## Results

In this study, we compared the BOLD responses to manual stroking and mechanical stimulation of the fingers within subject-specific, independently defined regions of interest (ROIs). The ‘mechanical stimulation’ run contained an odd-ball task to secure sufficient attention levels of the subjects. All subjects reported >90% of oddball stimuli.

An example of the labelling maps and BA ROI definitions is given in Fig 2 for a single subject. A clear gradient of D5-D4-D3-D2-D1 ordered responses in BA3b is in this slice found in the anterior-posterior direction within both BA3b (white region, see inset) and BA1 (brown region, see inset). In this example, no ordered somatotopy is visible within BA2, although such a somatotopic gradient does exist, with the middle finger, index finger and thumb clusters on more inferior slices.

All subjects had a digit ROI of multiple voxels within each BA in the ‘brush’ run. For two subjects, BA1 lacked an ROI for digit 4, for all other BA’s and subjects five digit ROIs could be defined. ROIs contained on average 140±15 voxels in BA3b, 120±15 voxels in BA1 and 120±20 voxels in BA2 (mean ± stderr over subjects and digits). Digit ROIs were somatotopically arranged (i.e. D5-D4-D3-D2-D1 sequentially) in all BA’s and subjects.

Within each stimulus type and BA, there were no significant differences between the different digit regions in terms of mean β values, mean t-values, maximum t-values or the number of voxels displaying statistically significant responses (1-way ANOVA, all p>0.4). Therefore, all values were grouped over the five digit regions. The mean β values, mean t-values, maximum t-values and number of voxels displaying statistically significant (p<0.05 FWE corrected) responses to stimulation of the ‘matching’ digit showed large differences between stimuli types and BAs (Table 2). All four measures were significantly different between ‘stroking’ and ‘mechanical stimulation’ runs (paired t-test, all p<0.001) in all three BA’s, with larger responses for stroking than for mechanical stimulation. In BA2, average β- and t-values in the brush-defined ROIs were significantly different from 0 only for the stroking and not for the mechanical stimulation.

**Table 2:** BOLD response measures in the three BAs for both stimulation methods.

Mean β value	BA 3b	BA 1	BA 2
Stroking	6.4 ± 0.4	6.2 ± 0.6	4.7 ± 0.4 ⱡ
Mechanical stimulation	1.8 ± 0.4 *	2.4 ± 0.4 *	0.5 ± 0.3 * ⱡ
**Mean t-value**			
Stroking	6.6 ± 0.3	5.9 ± 0.4	4.1 ± 0.4 ⱡ
Mechanical stimulation	2.1 ± 0.4 *	2.7 ± 0.4 *	0.6 ± 0.3 * ⱡ
**Maximum t-value**			
Stroking	14.7 ± 0.4	12.3 ± 0.7	10.2 ± 0.5 ⱡ
Mechanical stimulation	8.7 ± 0.6 *	9.2 ± 0.7 *	6.6 ± 0.5 *
**#voxels p<0.05 FWE**			
Stroking	88 ± 9	72 ± 11	47 ± 12
% of average ROI size	64%	59%	38%
Mechanical stimulation	24 ± 5 *	26 ± 6 *	4 ± 1 * ⱡ
% of average ROI size	17%	21%	3%

The mean β values, mean t-values, maximum t-values and number of voxels displaying statistically significant responses (p<0.05, FWE corrected) to the matching digit in BA 3b, BA 1 and BA 2. Values are presented as average over digits (5) and subjects (7) ± the standard error. A * indicates a significant difference (paired t-test, p<0.001) between the ‘mechanical stimulation’ and ‘stroking’ runs.

ⱡ indicates a significant difference with the corresponding value in BA3b (paired t-test, p<0.001). For reference, values on number of active voxels are also provided as a percentage of the average ROI size in the given BA.

There were no significant differences between responses in BA3b and BA1 for either stimulus type (paired t-tests, all p>0.1), while the mean β values and the mean and maximum t-values were significantly lower in BA2 than in BA1 and BA3b for both the ‘mechanical stimulation’ run and the ‘stroking’ run (p<0.05). The number of significant voxel at a threshold of p<0.05 FWE was significantly lower in the BA2 regions than in the BA1 and BA3b regions (p<0.05) for the ‘mechanical stimulation’ runs only. For the ‘stroking’ runs, the fraction of voxels above the p<0.05 FWE corrected threshold (Table 2) was relatively constant across BAs (38–64%), while for the ‘mechanical stimulation’ only 3% of the voxels in the BA2 ROI yielded a response significant at a threshold of p<0.05 FWE corrected.

The ROI definition also allows the extraction of beta values for cross-digit responses (see Table 1). If digit ROIs would have no overlap, no positive responses should be found in ‘adjacent’ or ‘distant’ digit ROIs. However, due to partial volume effects a small positive response in ‘adjacent’ digits should be expected. Also, ‘distant’ digit regions (for example the response of the D4 region during stimulation of D1) have been reported to yield negative responses, especially in BA3b [3]. Here, we extracted cross-digit responses for both ‘stroking’ and ‘mechanical stimulation’ runs, again based on the same, brush-defined ROIs.

**Fig 3:**
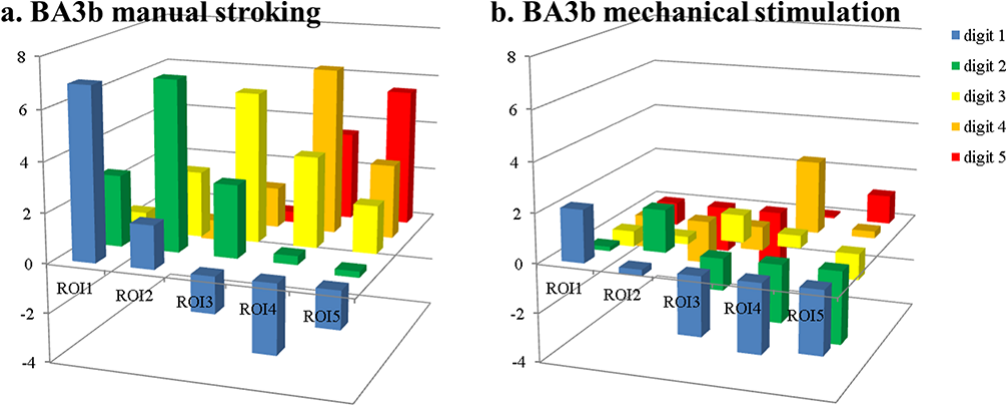
Beta values per digit ROI within BA3b. Beta values per digit ROI within BA3b for stroking (a) and mechanical stimulation (b). X-axes indicate the label of the ROI as defined from the ‘brush’ run; the y-axis defines the digit being stroked.

For all digit regions in BA3b, strongly positive responses were found for the stimulation of the ‘matching’ digit, confirming that the ROI definition based on the brush stimulus was appropriate. Cross-digit responses for the ‘stroking’ run (off-diagonal values in Fig 3A) matched the results found in previous work [3]. Especially big differences between manual stroking and mechanical stimulation were found for the ‘adjacent’ responses, which yielded an average beta value of 2.8±0.2* for stroking and -0.3±0.2 for mechanical stimulation. Values are reported here as mean±stderr over subjects and ROIs and * indicates a response significantly different from 0 (t-test, corrected for multiple comparisons, p<0.05). Stimulation of ‘distant’ digits yielded beta values of -0.4 ±0.3 and -1.9±0.3* for stroking and mechanical stimulation, respectively. Both in ‘adjacent’ and ‘distant’ ROIs the differences between stroking and mechanical stimulation responses were significant (paired t-tests, corrected for multiple comparisons, p<0.05).

**Fig 4:**
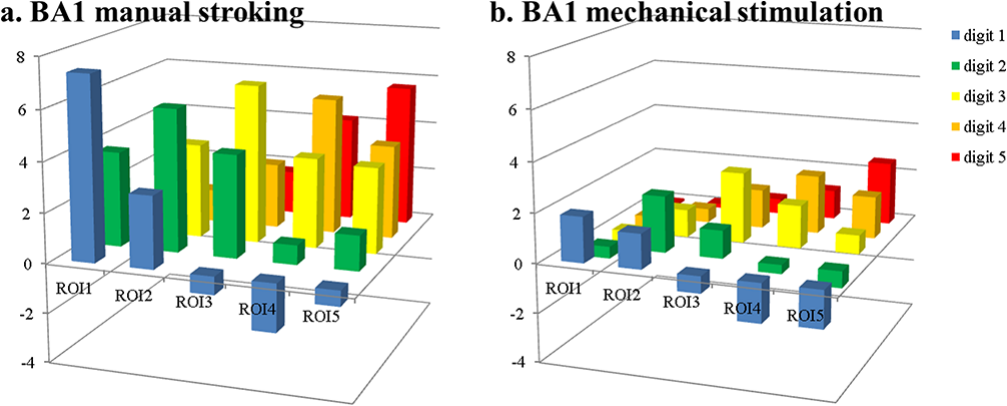
Beta values per digit ROI within BA1. Beta values per digit ROI within BA1 for stroking (a) and mechanical stimulation (b). X-axes indicate the label of the ROI as defined from the ‘brush’ run; the y-axis defines the digit being stroked.

In BA1, higher positive responses for ‘adjacent’ digits were found, now also for the ‘mechanical stimulation’ run, although ‘distant’ digits still yielded negative responses (Fig 4A and 4B). The average β-values were 3.6±0.3* and 1.2±0.3* for ‘adjacent’ digits in ‘stroking’ and ‘mechanical stimulation’ runs respectively and 0.9±0.3 and -0.4±0.3 for ‘distant’ digits. In BA1, as in BA3b, these differences between manual stroking and mechanical stimulation were significant (paired t-tests, corrected for multiple comparisons, p<0.05). These patterns of increased responses in ‘adjacent’ digit ROIs also confirm the higher overlap between digit regions in BA1 compared to BA3b reported previously [2,3].

**Fig 5:**
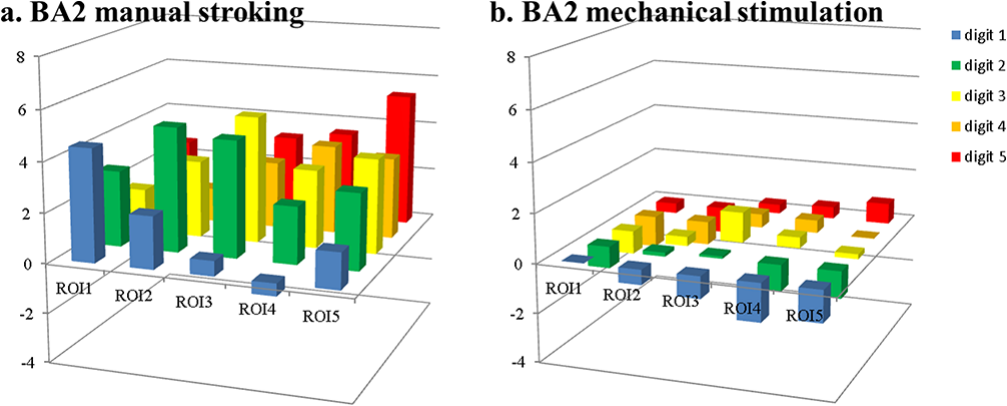
Beta values per digit ROI within BA2. Beta values per digit ROI within BA2 for stroking (a) and mechanical stimulation (b). X-axes indicate the label of the ROI as defined from the ‘brush’ run; the y-axis defines the digit being stroked.

In BA 2, even more overlap between digit ROIs was found, although the general somatotopic pattern was preserved, with highest responses for the ‘matching’ digits (average β = 4.7±0.4* for manual stroking and β = 0.5±0.3 for mechanical stimulation, see also Table 2), smaller responses for ‘adjacent’ digits (β = 3.2±0.4* and 0.0±0.3 for ‘stroking’ and ‘mechanical stimulation’ runs, respectively) and lowest responses for stimulation of ‘distant’ digits (β = 1.9±0.3* and -0.9±0.2*). Also in BA2, these differences were highly significant (paired t-tests, corrected for multiple comparisons, p<0.05). Although both ‘matching’ and ‘adjacent’ ROIs did not yield any significant responses in the ‘mechanical stimulation’ run, the highest responses were found for the ‘matching’ digits with both stimulation types (see also Fig 5A and 5B), confirming the validity of the brush-ROI definition in BA2.

**Fig 6:**
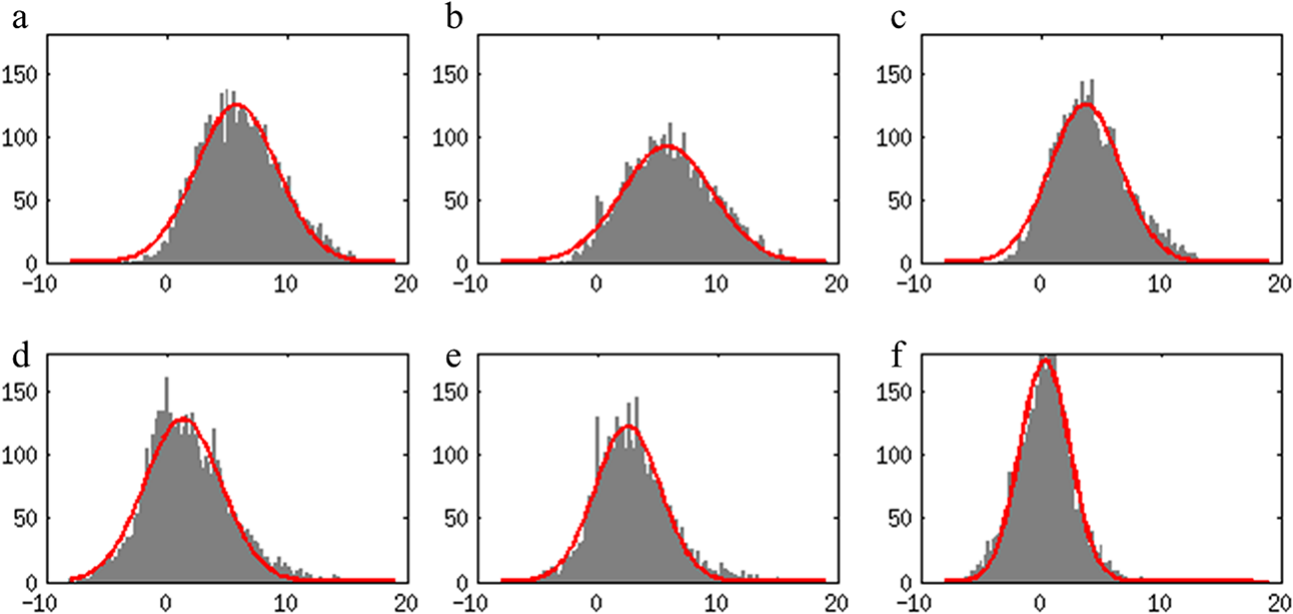
Histograms of t-values for stimulation of ‘matching’ digits. Voxel responses were grouped across subjects and digit ROIs to compare between BAs and stimulus type. a-c: stroking runs. d-f: mechanical stimulation runs. a/d: BA 3b, b/e: BA 1, c/f: BA2. The results of a t-distribution (Eq 1) fit to the distributions are shown overlaid in red. A small shoulder of high t-values can be seen in panel d; the responses for the mechanical stimulation run in BA3b.

The average changes observed in the ROIs could be due to either an overall change in the response to the stimulation or to a focusing of the BOLD response to a particular region within the digit ROIs for the mechanical stimulation. The distribution of t-values within the ROIs would differ, depending on the type of change. Therefore, we binned voxel responses to stimulation of the label-corresponding digit across digits and subjects to study their distributions (Fig 6). For both stroking and mechanical stimulation, t-value distributions closely resembled the fitted t-distribution curves, overlaid in red in Fig 6. The mean of the overall t-value distribution including all subjects’ data, μ_all_, was found at a t-value of 5.8, 5.8 and 3.7 for ‘stroking’ runs in BA3b, 1 and 2 respectively and at 1.3, 2.5 and 0.3 for ‘mechanical stimulation’ runs. The width of the overall distribution, σ_all_, was 3.4, 3.8 and 3.0 for ‘stroking’ runs again in BA3b, BA1 and BA2 and 3.2, 2.7 and 2.1 in ‘mechanical stimulation’ runs. The t-distribution functions fitted to individual subject distributions showed that the mean of the distribution was significantly higher for stroking than for mechanical stimulation in all BAs (paired t-tests, p<0.05 in all cases). The distribution was only in BA2 significantly different, (i.e. σ higher, p<0.05) but indicated a wider distribution for the ‘stroking’ runs than for the ‘mechanical stimulation’ runs. Of course, the scaling parameter S, reflecting the number of samples, did not differ between ‘stroking’ and ‘mechanical stimulation’ runs in any of the BAs. There was no indication of a skew in the distributions in either the ‘stroking’ or ‘mechanical stimulation’ runs.

**Fig 7:**
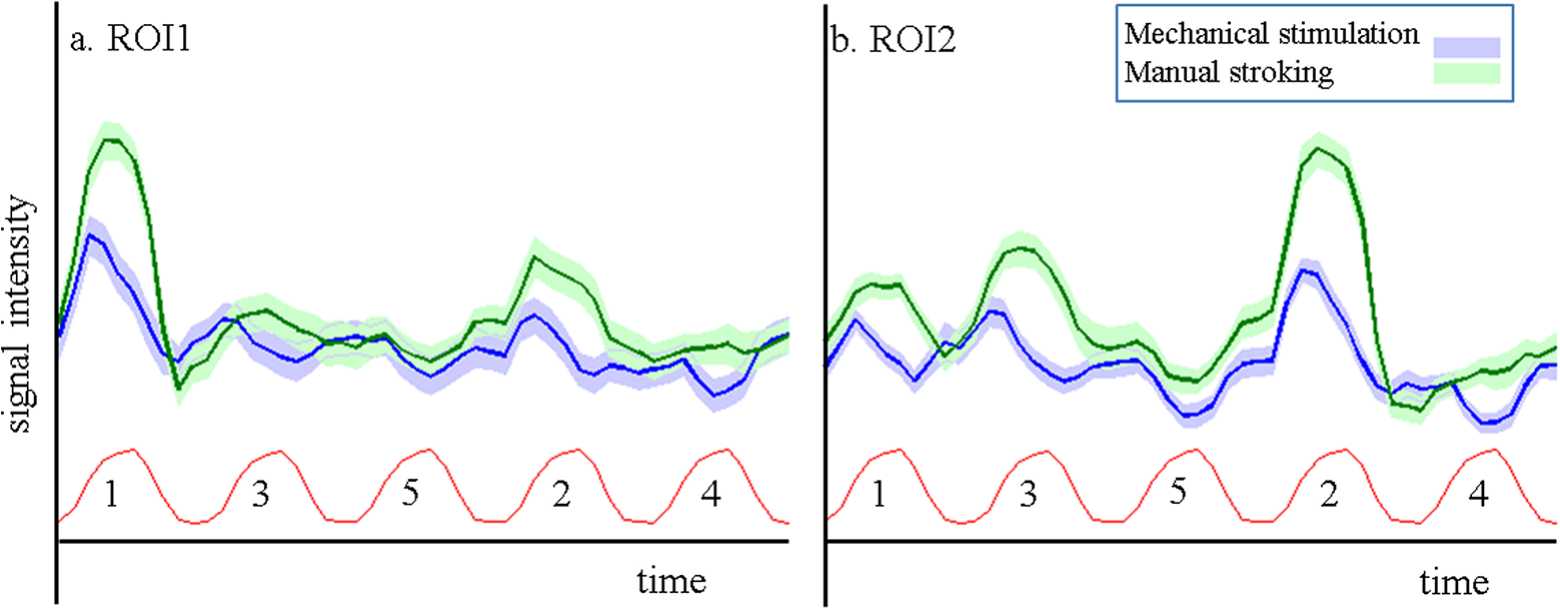
Average time courses. Average time courses of the ROIs for digit 1 and digit 2 in BA3b for manual stroking (green) and mechanical stimulation (blue) runs. Shaded regions indicate the standard error over subjects (7) and cycles (8). The SPM model function is shown in red under the time courses. Numbers indicate the digit being stroked in the corresponding block. Note the drops below baseline during stimulation of digit 5 (both ROIs, both runs) and digit 4 (both ROIs, ‘mechanical stimulation’ run).

Example time courses extracted from the smoothed and motion corrected data for two ROIs are shown in Fig 7, with the SPM model function shown as reference below. The stimulation of the ‘matching’ digit led in both ROIs to large positive responses, showing the good correspondence between the ‘brush’ runs used to define the ROIs and the ‘stroking’ and ‘mechanical stimulation’ runs. Stimulation of other digits led to either small positive responses, such as for the ‘adjacent’ digits D2 in ROI1, and D1 and D3 in ROI2, for both stroking and mechanical stimulation, or to negligible responses, such as for example D4 for manual stroking in ROI1, or to a visible reduction with respect to the baseline, such as for the ‘distant’ digit D5 for both stimuli and ROIs.

## Discussion

This study aimed at comparing the BOLD response within the primary somatosensory cortex to tactile stimulations delivered by different stimulation devices. In particular, we compared the response elicited by piezo-electric stimulators and manual stroking. The results showed that the BOLD responses in subject, digit and BA specific ROIs were larger for manual stroking than for mechanical stimulation in all tested brain regions. Cross-digit responses, such as the response to a middle-finger region during stroking of the thumb (see also Table 1), were found to be either small and positive for ‘adjacent’ digits or negative for stimulation of ‘distant’ digits, with overall more negative responses for mechanical stimulation than for manual stroking.

There are multiple possible causes for the differences in responses between the manual stroking and mechanical stimulation stimuli: the manual stroking response is more salient as it contains an element of motion and the perception of ‘body warmth’, whereas the mechanical stimulation is only a vibration, which might lead to responses from a smaller subpopulation of somatosensory nerve cells. During the manual stroking, another human is present in the scanner room (the experimenter), which might lead the subject to be more attentive. On the other hand, the subjects were performing a task during the ‘mechanical stimulation’ runs, and they were aware that their task performance was evaluated by the scanner operators. In addition, all ‘mechanical stimulation’ runs were performed first during the fMRI sessions for practical reasons, and first runs are typically assumed to lead to higher BOLD responses because later runs might be affected by fatigue and ennui [3]. Being touched by a human being might also elicit an emotional response, which could, in turn, lead to increased BOLD signal changes [43]. In terms of predictability, the manual stroking was performed as similar as possible to the mechanical stimulation. In fact, the stroking pattern resembled the mechanical stimulation frequency. However, minor irregularities in stroking onset were not avoidable and may make a difference for how the cutaneous inputs are processed in the primary sensory cortex.

Perhaps most importantly, the stroking stimulus affects a larger skin surface than the mechanical stimulation. If responses were limited solely to the brain regions corresponding to the finger tips [21], a spatially less extensive response would be found for mechanical stimulation. Therefore, we generated histograms of the BOLD responses within the digit ROIs to test for the possibility that only a sub-section of the ROI responded to the mechanical stimulation, whereas the entire ROI responded to manual stroking. Because the ROIs were defined based on the brush-stroking data, which also stimulated the full two distal phalanges of each digit, this seemed a reasonable hypothesis. However, distributions of t-values for mechanical stimulation were no more skewed than for manual stroking for any of the 3 anatomical ROIs in Fig 6. Thus, the surface extent of stimulated skin is unlikely to be the cause of the large differences seen in responses to the manual stroking and mechanical stimulation.

The example time courses (Fig 7) extracted from the ROIs showed the pattern of reducing β values for increasing digital distance to the stimulated finger. However, when comparing the time courses to the β values averaged over same region, it becomes clear that the temporal dynamics of the hemodynamic response also influence the obtained β value. For example, in the ROI assigned to the thumb in BA3b (Fig 7A), extracted β values for manual stroking of D1, D2, D3, D4 and D5, respectively were 6.9, 2.9, 0.8, -0.2 and 0.1 (Fig 3A, leftmost column). These values match well with the qualitative expectation from the green curve in Fig 7A. However, for the same region, mechanical stimulation yielded β values of 2.1, -0.2, -0.6, -1.4 and -1.0, which is more negative than what might be expected from visual inspection of the blue time course. The negative responses, i.e. drops of signal below that at baseline (rest), during stimulation of D4 and D5 is clear. But both stimulation of D2 and D3 appears to lead to very short, small but positive signal changes in the thumb ROI (Fig 7A). Negative β values are also found in the index finger ROI (Fig 7B) for mechanical stimulation of D1 and D3, although the time courses indicate short positive deviations from baseline. That these nevertheless result in negative β values is due to the fast return to baseline of the BOLD response, much quicker than the modeled hrf as defined from the 10s stimulus length. This faster return to baseline is also evident in the responses to mechanical stimulation of the ‘matching’ digit (D1 in Fig 7A, D2 in Fig 7B), although the same effect is not visible in the response curves to manual stroking. These faster returns to baseline suggest faster habituation to the mechanical stimulation than to the manual stroking, even though the task required subjects to attend to the stimulus during the entire 10 second block, and their performance suggests they attended the stimuli continuously. Apart from the task, stimuli were presented in a pulsed fashion (500 ms ON, 500 ms OFF) to reduce habituation effects. It is interesting to note that, while responses of ‘adjacent’ digits were short and positive, stimulation of ‘distant’ digits did lead to solid negative responses, with clear drops below baseline in the time courses, which were in fact larger in the ‘mechanical stimulation’ runs. Therefore, habituation can only partially explain the differences found between mechanical stimulation and manual stroking responses.

It is interesting to note that these drops below baseline in ‘distant’ digit ROIs, whether for mechanical stimulation or for manual stroking, will increase the contrast if a relative contrast (i.e. thumb vs middle finger) is used rather than stimulation of a single digit vs rest, as done here. This may partially explain why earlier approaches [26,44], including the travelling wave approach [14,21] and a pattern-recognition based analysis [20] successfully identified digit representations in the primary somatosensory cortex based on mechanical stimulation.

The most likely explanation for the large difference between responses to manual stroking and mechanical stimulation is a combination of all the aforementioned points, leading to overall higher salience of the manual stroking stimulus and thus to higher BOLD responses. The higher salience, combined with the larger number of features associated to stroking compared to simple vibrations, might affect responses in BA2 more than in BA3b and BA1 because of the more integrative nature of BA2 [45]. This could reflect why only a minimal proportion of the BA2 ROIs displayed p<0.05 FWE responses to mechanical stimulation while p<0.05 FWE responses were found in a relatively constant fraction of the ROIs in ‘stroking’ runs (Table 2). As habituation is likely to be the cause of the earlier return to baseline in the ‘adjacent’ ROIs, a more irregular temporal stimulation pattern may result in more sustained responses.

Of course, when selecting a somatosensory stimulus for somatotopic mapping, there are further factors to consider besides stimulus efficacy. The choice might be determined by the availability of stimulation equipment; the price of new equipment if it is to be acquired; limited available space in the magnet bore, especially in high-field scanners; and feasibility of accessing the required body part–for example for the face, manual stroking would be highly impractical and the presence of a piezo-electric device inside the rf-coil would deteriorate MR data quality. In such a case, airpuffs or a remotely operated brush system would probably be preferable. In this study, the comparison is limited to two types of somatosensory stimuli: the widely used piezo-electric mechanical stimulator and the manual stroking stimulus, which were evaluated in terms of the measured BOLD responses in independently defined ROIs.

## Conclusion

Mechanical stimulation and manual stroking were compared in a somatotopic mapping procedure based on ultra-high field, high resolution fMRI. Manual stroking led to significantly higher BOLD responses in all anatomy-functionally defined ROIs in BA3b, BA1 and BA2. This might be due to the more salient sensation of touch by a human finger. Histogram analysis of voxel-wise responses did not show more focussed responses for either stimulus type.

The use of mechanical stimulation in combination with an odd-ball task did lead to the detection of more negative BOLD responses in non-stimulated digits. Positive responses following mechanical stimulation tended to return to baseline earlier than responses to manual stroking in the same ROIs, possibly due to faster habituation to the mechanical stimulus.

Overall, these differences suggest that while mechanical stimulation and manual stroking elicited activations that were highly co-localized within BA 3b and 1, the higher BOLD signal intensity induced by the manual stroking makes it a more powerful stimulus for somatotopic mapping. The use of a manual stroking paradigm will be especially valuable in cases where a somatotopic subdivision of BA 2 is desired.
